# Identifying a human signal in the North Atlantic warming hole

**DOI:** 10.1038/s41467-020-15285-x

**Published:** 2020-03-24

**Authors:** Rei Chemke, Laure Zanna, Lorenzo M. Polvani

**Affiliations:** 1 Department of Applied Physics and Applied Mathematics, Columbia University, New York, NY USA; 2 Atmospheric, Oceanic and Planetary Physics, Department of Physics, University of Oxford, Oxford, UK; 3 Courant Institute of Mathematical Sciences, New York University, New York, USA; 4 Department of Earth and Environmental Sciences, and Lamont-Doherty Earth Observatory, Columbia University, Palisades, NY USA

**Keywords:** Climate change, Attribution, Climate and Earth system modelling

## Abstract

North Atlantic sea surface temperatures have large climate impacts affecting the weather of the Northern Hemisphere. In addition to a substantial warming over much of the North Atlantic, caused by increasing greenhouse gases over the 21st century, climate projections show a surprising region of considerable future cooling at midlatitudes, referred to as the North Atlantic warming hole. A similar pattern of surface temperature trends has been observed in recent decades, but it remains unclear whether this pattern is of anthropogenic origin or a simple manifestation of internal climate variability. Here, analyzing state-of-the-art climate models and observations, we show that the recent North Atlantic warming hole is of anthropogenic origin. Our analysis reveals that the anthropogenic signal has only recently emerged from the internal climate variability, and can be attributed to greenhouse gas emissions. We further show that a declining northward oceanic heat flux in recent decades, which is linked to this surface temperature pattern, is also of anthropogenic origin.

## Abstract (web summary)

Most of the North Atlantic has seen strong increase in surface temperatures in recent decades, except for one region, which has been called the North Atlantic warming hole. Here, the authors employ detection and attribution techniques to show that this temperature pattern in recent decades is being caused by anthropogenic greenhouse gas emissions.

## Introduction

Identifying a human fingerprint in the climate system is an integral part in the line of evidence of the effects of human activity on climate. Given the large variability of the climate system, detecting such fingerprints involves separating the internal variability of the system from its forced response to anthropogenic emissions. Here we focus on identifying a human impact on North Atlantic sea surface temperatures (SSTs), since they play a major role in the climate of the Northern Hemisphere^1–6^.

Unlike previous studies which have focused on linking the SST patterns in the North Atlantic to changes in the oceanic circulation^3,7–16^, we here perform a formal detection-attribution analysis in order to identify the human fingerprint in North Atlantic SST over recent decades.

## Results

### The North Atlantic warming hole

**Fig. 1:**
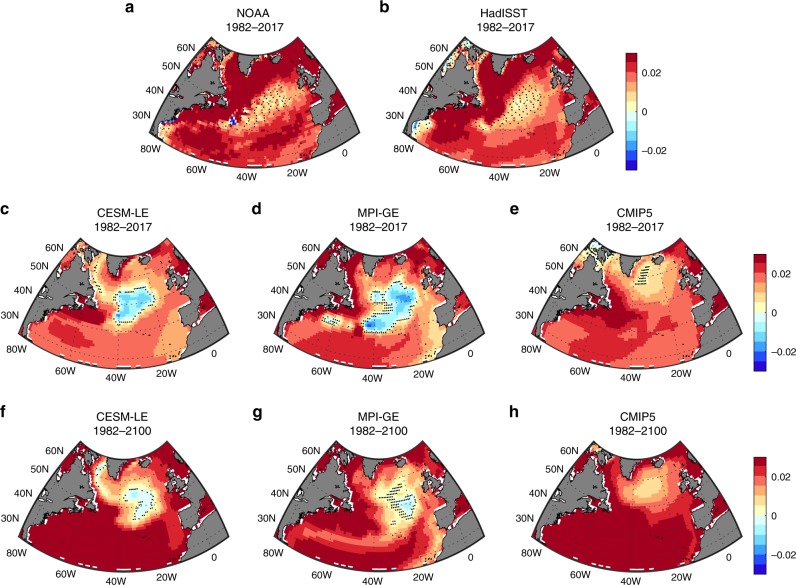
Annual mean sea surface temperature trends (Kyr^−1^). The 1982–2017 sea surface temperature trends from satellite-based observations in **a** NOAA, and **b** HadISST. The 1982–2017 simulated sea surface temperature trends from the mean of the **c** Community Earth System Model Large Ensemble (CESM-LE), **d** Max Planck Institute for Meteorology Grand Ensemble (MPI-GE), and **e** Coupled Model Intercomparison Project Phase 5 (CMIP5). The 1982–2100 simulated sea surface temperature trends from the mean of **f** CESM-LE, **g** MPI-GE, and **h** CMIP5. The small black crosses show where the sea surface temperature trends are statistically insignificant based on a Student *t*-test (*p*-value > 0.05).

We start by illustrating the recent (1982–2017) North Atlantic SST trends (Fig. 1). Two SSTs datasets, which combine satellite and in-situ measurements (the high-resolution NOAA SST^17^ and HadISST^18^, Methods), show stronger warming at high and low latitudes than at midlatitudes, resulting in the so-called North Atlantic Warming Hole pattern^6,7,10–12,14,15,19^ (Fig. 1a, b). We focus here on the most recent 36 years in order to include at least one high-resolution observational product (the high-resolution NOAA SST is only available since 1982), and because this warming hole pattern in the North Atlantic is absent prior to the 1980’s (Supplementary Fig. 1). Recently, a similar warming hole pattern in the North Atlantic was found using a single simulation of high-resolution global circulation model run with doubled CO_2_ concentrations^14^. The analysis of a single idealized forced simulation does not allow for the attribution of the recent SST patterns to greenhouse gas emission, as the role of internal variability and of the recent changes in both natural and anthropogenic forcing agents cannot be assessed. As a first step to determining if the observed trends constitute the system’s forced response to anthropogenic emission, we compare the observed SST trends with the 1982–2017 mean trends from two ensembles of model simulations: the Community Earth System Model 40-members Large Ensemble (CESM-LE)^20^ and the Max Planck Institute Earth System Model 100-members Grand Ensemble (MPI-GE)^21^ (Methods). Recall that all simulations are subjected to the same Historical forcing from 1850 to 2005, and the same Representative Concentration Pathway 8.5 (RCP8.5) through 2100. To the degree that the models realistically capture the behavior of the climate system, and provided the ensemble size is sufficiently large, the mean of the ensemble members represents the system’s response to external forcings, as the internal variability is eliminated by averaging across the members of the ensemble (Methods).

The forced response of the SST in the mean of the CESM-LE and MPI-GE (Fig. 1c, d) show a pattern similar to the observed one, with warming at high and low latitudes and weak cooling trend at midlatitudes^15^. The similarity of the forced response in CESM-LE and MPI-GE suggests that the smaller ensemble size of the CESM-LE does not affect the forced signal (Supplementary Fig. 2). One reason for the stronger cooling—and its different shape—in the ensemble means compared to the observations might be that internal variability obscures part of the system’s forced response (ensemble members exhibit considerable differences in the shapes and amplitudes of the warming hole, Supplementary Fig. 3). But the strong cooling in the ensemble means could also be due to biases in these two models’ physical or numerical formulations (e.g., biases in external forcing or subgrid parameterizations) that prevent them from accurately simulating the behavior of the climate system. To address this we also analyze 35 model simulations from the Phase 5 of the Coupled Model Intercomparison Project^22^ (CMIP5, Methods). These simulations also run under the Historical and RCP8.5 scenarios, but each simulation is carried out with a different model. Thus, the CMIP5 multi-model mean removes part of the internal variability, and also part of the spread due to the different model formulations, assuming no systematic biases across all models (Supplementary Fig. 4). Figure 1e shows the 1982–2017 SST trends from the mean of the CMIP5 ensemble, which also shows a similar warming hole pattern. Unlike for CESM-LE and MPI-GE, the warming hole in the CMIP5 mean is weaker and located poleward owing to the averaging across models with different patterns (potentially related to the different locations of deep water formation and strength of the oceanic circulation across the models^13^). Nonetheless, a warming hole pattern is clearly present in the CMIP5 mean.

### Detecting a human signal in the North Atlantic

**Fig. 2:**
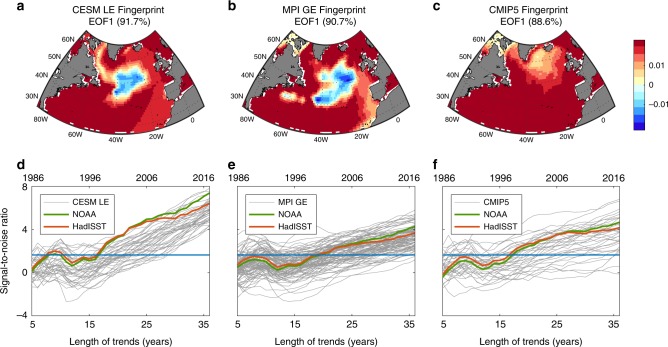
Fingerprints and signal-to-noise analysis. The leading mode (fingerprint) of the 1982–2017 North Atlantic sea surface temperature anomalies in the **a** Community Earth System Model Large Ensemble (CESM-LE), **b** Max Planck Institute for Meteorology Grand Ensemble (MPI-GE), and **c** Coupled Model Intercomparison Project Phase 5 (CMIP5). The percentage in each panel represents the variability explained by each mode. Signal-to-noise ratio for North Atlantic sea surface temperature as a function of the length of trends (and corresponding last year of trend) since 1982 using the fingerprint and noise from **d** CESM-LE, **e** MPI-GE, and **f** CMIP5. The signal in the green and orange lines is calculated using the satellite-based observations in NOAA, and HadISST, respectively. The signal in the gray lines in each panel is calculated using each realization in the CESM-LE, MPI-GE, and CMIP5 datasets. The blue horizontal line marks the 5% significance threshold.

Given the similarity between the observed and forced model-mean SST patterns, can one formally detect the externally forced signal in the observed SST? To answer that we follow previous studies^23–26^ and use a standard fingerprint detection analysis over the North Atlantic region, based on computing the signal-to-noise ratio (SNR) (Methods). First, the externally forced fingerprint is defined using the leading empirical orthogonal function (EOF) of the 1982–2017 North Atlantic SST anomalies (defined relative to the 1982–2017 climatology) in the mean of each ensemble (Fig. 2a–c). These fingerprints explain most of the SST variability (~90%), and are very similar to the forced SST trends in the respective model ensembles (Fig. 1c–e). Second, the signal is defined by computing trends of increasing lengths of the projection of the observed SST anomalies onto these fingerprints (i.e., the spatial sum, at each year, of the product of the observed anomalies and each fingerprint, Methods). The fact that these projections increase with time indicates that the observed SST become progressively more similar to the externally forced fingerprints (Supplementary Fig. 5). The noise is defined in a similar way, but using the SST from the preindustrial control runs (where the anthropogenic forcing is absent, and only the internal variability of the climate system is present) rather than the observed SST. The externally forced fingerprint is then detected in the observation once the signal exceeds the 5% significance threshold relative to the noise (Methods). This SNR calculation can be done not only for the observations but also for each realization in the three ensembles.

Figure 2d–f shows the SNR calculated from observations (green and orange lines) and from each ensemble member in the CESM-LE, MPI-GE, and CMIP5 (gray lines), along with the 5% significance threshold (horizontal blue lines). As in previous studies^24,25^, the increase in SNR drops after 1991 due to anomalous cold temperatures after that year, perhaps associated with the volcanic eruption of Mt. Pinatubo. Thereafter, the SNR monotonically increases and the externally forced fingerprints from each of the ensembles are clearly detectable in the two satellite-based observations. These fingerprints emerge from the internal variability of the system around the year 2000. Furthermore, we emphasize that the fingerprint is detectable in nearly all individual members of the CESM-LE and MPI-GE after the year 2000, implying that these models well capture the observed SST pattern changes. Given the similarity of the forced fingerprints in CESM-LE and MPI-GE, the more rapid increase in SNR in CESM-LE than in MPI-GE implies that the larger variability in the MPI-GE preindustrial run delays the emergence of the forced signal: one standard deviation of the noise from MPI-GE is 35% larger than one standard deviation of the noise from CESM-LE. For the CMIP5, the larger spread of SNR across the models, when compared to the CESM-LE and the MPI-GE spreads, illustrates the effects of the different models’ formulation on the emergence of the forced SST pattern.

**Fig. 3:**
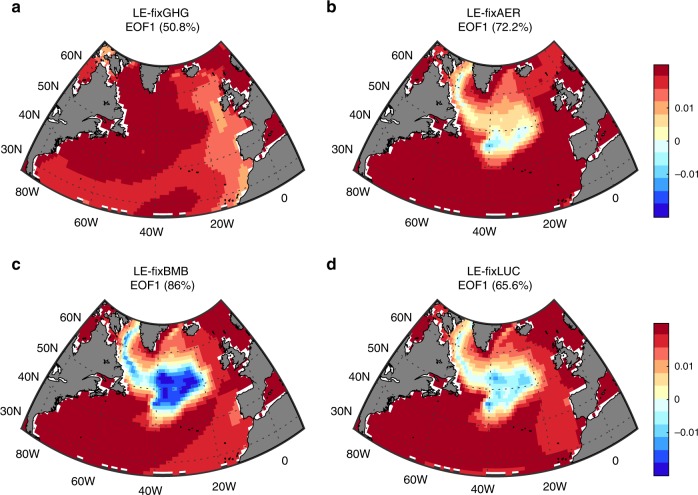
Attribution analysis for North Atlantic sea surface temperature. The leading mode (fingerprint) of the 1982–2017 North Atlantic sea surface temperature anomalies in the four Community Earth System Model (CESM) ensembles with **a** fixed greenhouse gases (LE-fixGHG), **b** fixed aerosols (LE-fixAER), **c** fixed biomass burning (LE-fixBMB), and **d** fixed land use/change (LE-fixLUC). The percentage in each panel represents the variability explained by each mode.

Are the externally forced fingerprints that we detect in the observations anthropogenic or natural? To answer this question we make use of four large ensembles of model simulations nearly identical to the CESM-LE, but with one forcing agent kept constant: greenhouse gases (LE-fixGHG), aerosols (LE-fixAER), biomass burning (LE-fixBMB) and land use/land change (LE-fixLUC) (Methods). Figure 3a–d shows the fingerprints of the 1982–2017 SST anomalies in each of these ensembles. When the greenhouse gases are fixed, the CESM-LE fingerprint disappears (compare Figs. 3a and 2a). In contrast, when fixing the aerosols, biomass burning or land use/land change the CESM-LE fingerprint persists, attesting that the detectable fingerprints in the observation can be attributed to greenhouse gas emissions. One is then led to ask how this SST pattern will evolve over the 21st century, when greenhouse gases are projected to constantly increase. Not surprisingly, the warming hole pattern in the three ensembles persist through the end of the 21st century^7,9,13,15^ (Fig. 1f–h), confirming that it is indeed related to the ongoing anthropogenic emissions.

### The underlying mechanism of a human signal in North Atlantic

Finally we ask, can one also attribute the mechanisms that are responsible for the formation of the forced SST fingerprint to greenhouse gas emissions? To answer this we first quantify the relative importance of the processes that affect the SST pattern. We analyze the CESM-LE mixed-layer temperature tendency equation for the North Atlantic midlatitudes over the warming hole region (20°W – 40°W and 45°N – 55°N). We use the CESM-LE, rather than the MPI-GE or CMIP5, since the model output needed for calculating the mixed-layer temperature equation is only available for that ensemble. According to the mixed-layer temperature tendency equation (Eq. (1), Methods^27^), four terms govern its temporal behavior: air–sea heat fluxes, zonal and meridional advection, and vertical heat transfers.

**Fig. 4:**
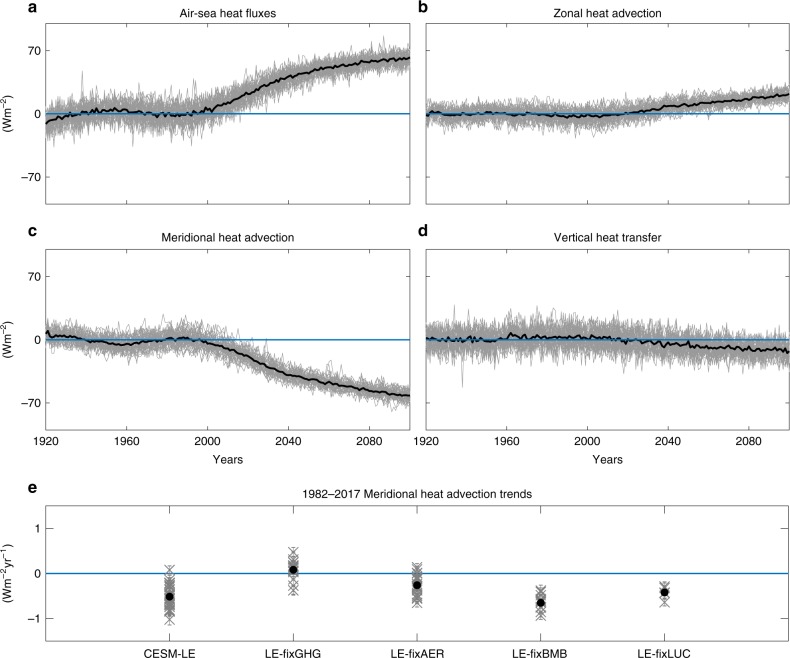
Attribution analysis for the North Atlantic sea surface temperature fingerprint mechanism. The Community Earth System Model Large Ensemble (CESM-LE) annual mean mixed-layer temperature tendency equation anomalies (Wm^−2^, Eq. (1), Methods), relative to the 1920–1960 period, averaged over the warming hole region (see text); **a** Surface air-sea heat fluxes. **b** Zonal heat advection. **c** Meridional heat advection. **d** Vertical heat transfers. The gray and black lines represent all CESM-LE members and their mean, respectively. **e** The 1982–2017 meridional heat advection trends in five CESM ensembles with all forcing agents (CESM-LE), fixed greenhouse gases (LE-fixGHG), fixed aerosols (LE-fixAER), fixed biomass burning (LE-fixBMB), and fixed land use/change (LE-fixLUC).

The annual mean time evolution of the four terms governing the mixed-layer temperature tendency equation for the North Atlantic midlatitudes shows that throughout the 21st century the main balance in the CESM-LE simulations is between air–sea heat fluxes and meridional heat advection (Fig. 4a–d). An increasing trend in air-sea heat flux (Fig. 4a), which is mostly driven by a reduction in latent heat fluxes, acts to warm the SST. However, since the early 90’s, meridional heat advection drives a cooling trend by transferring less heat to midlatitudes (Fig. 4c). This cooling trend opposes and—in some locations overcomes—the warming by air–sea heat fluxes resulting in the North Atlantic warming hole. This shows that the CESM-LE fingerprint (Fig. 2a) is due to a decline in meridional heat advection, confirming previous studies^3,7–16^. The other two terms (zonal and vertical heat transfers) do not contribute to the formation of the warming hole in the CESM-LE (Fig. 4b, d).

Lastly, to examine whether the recent decline in meridional heat advection can be attributed to greenhouse gas emissions, we compare its 1982–2017 trends in the CESM-LE to the trends in LE-fixGHG, LE-fixAER, LE-fixBMB, and LE-fixLUC in Fig. 4e. As for the SST fingerprint in Fig. 3, the recent meridional heat advection decline is again mostly associated with greenhouse gas emissions: in comparison to the other forcings, only fixing greenhouse gases results in a lack of forced decline in meridional heat advection. This mechanism is consistent with a weakening of the ocean circulation found in future climate projections^8,13^.

## Discussion

In conclusion, we note that recent and projected North Atlantic warming hole is, at first glance, a surprising feature, since one naively expects SSTs to warm with increasing greenhouse gases. What we have shown here is that the SST pattern in the North Atlantic that includes reduced warming at midlatitudes, relative to other latitudes, is indeed caused by anthropogenic emissions of greenhouse gases, and is related by changes in the oceanic circulation. And, if the human influence on North Atlantic SST documented in this study continues in coming decades, it is likely to further impact the climate of the many regions of the Northern Hemisphere, including the U.S., Europe and parts of Africa^1–6^.

## Methods

### Observations

Two data sets of sea surface temperature, which combine satellite and in-situ measurements, are used: the high-resolution NOAA SST^17^ and the Hadley Centre HadISST^18^. The NOAA SST is derived using the Advanced Very High Resolution Radiometer (AVHRR). The data is produced daily with 0.25° grid resolution since 1982. The HadISST is taken from the Met Office Marine Data Bank and is produced monthly with 1° grid resolution since 1870.

### Large ensembles

Two ensembles of ocean-atmosphere coupled model simulations are used; the Community Earth System Model 40-members Large Ensemble (CESM-LE)^20^, and the Max Planck Institute for Meteorology 100-members Grand Ensemble (MPI-GE)^21^. The CESM-LE is conducted using the CESM1^28^ and includes 40 simulations (members) running from 1920 to 2100. From 1920 to 2005 all members are subjected to the Historical forcing, and from 2006 to 2100 to the Representative Concentration Pathway 8.5 forcing (RCP8.5). The MPI-GE is conducted using the Max Planck Institute Earth System Model (MPI-ESM1.1) and includes 100 simulations (members) running from 1850 to 2100 under the same Historical and RCP8.5 scenarios. For disentangling the forced response and the internal climate variability each member in the ensembles is initialized with different initial conditions. The mean of each ensemble averages out the internal variability, thus represents the forced response of the system. Additional multi-century preindustrial control runs, using constant 1850 forcing, are used for each model, where the coupled CESM1 runs for 1800 years, and the MPI-ESM for 2000 years. The constant forcing in these simulations enables accounting only the internal climate variability.

Finally, for attributing the detectable fingerprint to a specific forcing agent we make use of four ensembles that are identical to the CESM-LE, but each ensemble excludes the time evolution of one forcing agent: greenhouse gases (LE-fixGHG, include 20 members), aerosols (LE-fixAER, include 20 members), biomass burning (LE-fixBMB, include 15 members), and land use/land change (LE-fixLUC, include 5 members).

### CMIP5

We also analyze 35 models that participate in the Coupled Model Intercomparison Project Phase 5^22^ (CMIP5), and select the ’r1i1p1’ member between 1850 and 2100 with the Historical and RCP8.5 scenarios (Supplementary Table 1). The last 200 years of each model’s preindustrial control run are used for the fingerprint analysis, as discussed below.

### Fingerprint analysis

To assess whether a forced fingerprint is statistically identifiable in the observations we follow previous studies^23–26^, and employ a standard fingerprint detection method using a signal-to-noise ratio approach. In this approach, one quantifies that the pattern similarity between the observations and the fingerprint increases with time (the signal) and emerges out of the internal variability of the system (the noise). This analysis is conducted over the North Atlantic region, shown in the figures in the manuscript, between 83°W – 8°E and 26°N − 67°N. First, the forced fingerprint (FP) is defined as the leading empirical orthogonal function (EOF) of the 1982–2017 annual mean North Atlantic SST anomalies (relative to the 1982–2017 climatology) in the mean of each ensemble. As discussed above, the mean of the ensembles averages out the internal variability, thus accounts only the forced response of the system. Although we here analyze only the observed period, the 1982–2017 fingerprints based on the ensembles continue throughout the 21st century (Supplementary Fig. 6).

Second, the annual mean observed SST’s anomalies (OB) are projected onto the fingerprints yielding a time series, ${\rm{S(t)}}=\mathop{\Sigma }\nolimits_{{\rm{x}}}{\rm{OB(x,t)}}\cdot {\rm{FP(x)}}$, where *x* represents the grid points and *t* the years. All fields used in the analysis are regridded to the same 1° × 1° grid, and are area-weighted by multiplying each field by the square root of the cosine latitude. Supplementary Figure 5 shows these time series using the two satellite-based SSTs and the three model fingerprints (the three ensembles). The “signal” is then defined by calculating trends over different lengths in each projection. The trends are first calculated over 5 years (from 1982 to 1986) and then over consecutive lengths of trends (from 1982 to 1987, 1988...2017).

Finally, to determine whether the forced fingerprint is statistically identifiable in the observations, the signal (the trends with different lengths) is compared to Gaussian distributions of trends with the same lengths from a preindustrial control run. The sole presence of internal variability of the climate system in the control run allows one to verify whether the fingerprint emerged out of the internal variability. The trends from the control run are calculated in the same manner as in the observations, but by projecting the annual mean SST’s anomalies from the control run onto the fingerprints. This is done for the CESM-LE and MPI-GE using all overlapping trends from all years from their control runs, and for the CMIP5, following previous studies^24–26^, by concatenating the last 200 years of the control run of each model (total of 7000 years). In each control run, the mean value of the annual mean SST’s is first removed, and the data is detrended at each grid point in order to avoid any drifts. The noise is then defined as one standard deviation (*σ*) of the distribution of each length of trends from the control run of each data set. The forced fingerprint is statistically identifiable in the observations once the signal exceeds a significance threshold (*p*-value) relative to the noise of 5% (1.645*σ*, using one-sided Student’s *t*-test).

### Mixed layer temperature equation

The mixed layer temperature equation^27^ can be written as follows,

1 $$\begin{array}{lll}\rho {c}_{p}h\frac{\partial {\rm{SST}}}{\partial t}={Q}_{{\rm{air}}}-\rho {c}_{p}h\left({u}_{g}+{u}_{\tau }\right)\frac{1}{a\cos \theta }\frac{\partial {\rm{SST}}}{\partial \phi }-\rho {c}_{p}h\left({v}_{g}+{v}_{\tau }\right)\frac{1}{a}\frac{\partial {\rm{SST}}}{\partial \theta }\\ \hskip -68pt- \, \rho {c}_{p}hw\frac{\partial {\rm{SST}}}{\partial z}+\rho {c}_{p}hw{Q}_{{\rm{tur}}}\end{array}$$

where, *t* is time, *ϕ* and *θ* are longitude and latitude, respectively, *a* = 6371 × 10^3^ m is Earth’s radius, *ρ* = 1027 kgm^−3^ is sea-water density, *c*_*p*_ = 3985 Jkg^−1^K^−1^ is the ocean specific heat capacity, *h* = 100 m is a reference North Atlantic mixed layer depth^29^, *Q*_air_ represents the air–sea heat fluxes (i.e., radiative longwave and shortwave fluxes, as well sensible and latent heat fluxes), *u*_*g*_ and *v*_*g*_ are the zonal and meridional geostrophic velocities, respectively, and *u*_*τ*_ and *v*_*τ*_ are the zonal and meridional Ekman velocities, respectively. The geostrophic velocities are calculated based on geostrophic balance, ${V}_{g}{\boldsymbol{=}}\frac{g}{f}{\boldsymbol{\times }}{\nabla }_{h}{\rm{SSH}}$, where *V*_*g*_ is the horizontal geostrophic velocity vector, *g* = 9.81 ms^−2^ is gravity, $f=2\Omega \sin \theta$ is the Coriolis parameter, where *Ω* = 7.292 × 10^−5^ s^−1^ is Earth’s rotation rate and SSH is the sea surface height. The Ekman transport in the mixed layer is calculated as $h{V}_{\tau }=\int {V}_{\tau }dz=-\frac{1}{\rho f}\times {{\tau }}$, where *V*_*τ*_ is the horizontal Ekman velocity vector and *τ* is the wind stress. The two rightmost terms on the right hand side of Eq. (1) represent any vertical heat transfers (advection, entrainment at the base of the mixed layer and turbulent mixing), where *w* is the vertical velocity and *Q*_tur_ is vertical heat transfer through turbulent mixing. Because the last two terms (vertical heat transfers) are not available, and in order to close the budget, these terms are calculated as the residual between the SST tendency (left hand side of Eq. (1)) and the air–sea heat fluxes and horizontal advection terms.

## Supplementary information

Supplementary Information

## Data Availability

The data used in the manuscript is publicly available from NOAA SST (https://www.esrl.noaa.gov/psd/), HadISST https://www.metoffice.gov.uk/hadobs/hadisst/), CESM-LE (http://www.cesm.ucar.edu/), MPI-GE (https://www.mpimet.mpg.de/en/grand-ensemble/), and CMIP5 data (https://esgf-node.llnl.gov/projects/cmip5/).

## Code availability

Any codes used in the manuscript available upon request from rc3101@columbia.edu.
