# Supplementary material for: Identifying a human signal in the North Atlantic warming hole
Source: Nat Commun. 2020 Mar 24;11:1540. doi: 10.1038/s41467-020-15285-x (PMC7093405; doi:10.1038/s41467-020-15285-x)
Supplement: Supplementary file 1 — Supplementary Information [file 41467_2020_15285_MOESM1_ESM.pdf]

Supplementary Material for: Identifying a human signal in the North Atlantic warming hole

Rei Chemke[1], Laure Zanna[2,3] & Lorenzo M. Polvani[1,4]

[1] *Department of Applied Physics and Applied Mathematics, Columbia University, New York, NY, USA*

[2] *Atmospheric, Oceanic and Planetary Physics, Department of Physics, University of Oxford, Oxford, UK*

[3] *Courant Institute of Mathematical Sciences, New York University, New York, USA*

[4] *Department of Earth and Environmental Sciences, and Lamont-Doherty Earth Observatory, Columbia University, Palisades, NY, USA*

Table 1: List of the 35 models from the Coupled Model Intercomparison Project Phase 5 analyzed in this study.

	**Model**	**Modeling Center**
1	ACCESS1.0	CSIRO (Commonwealth Scientific and Industrial Research Organisation, Australia), and BOM (Bureau of Meteorology, Australia)
2	ACCESS1.3	CSIRO (Commonwealth Scientific and Industrial Research Organisation, Australia), and BOM (Bureau of Meteorology, Australia)
3	bcc-csm1-1	Beijing Climate Center, China Meteorological Administration
4	bcc-csm1-1-m	Beijing Climate Center, China Meteorological Administration
5	BNU-ESM	College of Global Change and Earth System Science, Beijing Normal University
6	CanESM2	Canadian Centre for Climate Modelling and Analysis
7	CCSM4	National Center for Atmospheric Research
8	CESM1-BGC	National Science Foundation, Department of Energy, National Center for Atmospheric Research
9	CESM1-CAM5	National Science Foundation, Department of Energy, National Center for Atmospheric Research
10	CMCC-CESM	Centro Euro-Mediterraneo per I Cambiamenti Climatici
11	CMCC-CM	Centro Euro-Mediterraneo per I Cambiamenti Climatici
12	CMCC-CMS	Centro Euro-Mediterraneo per I Cambiamenti Climatici
13	CNRM-CM5	Centre National de Recherches Meteorologiques / Centre Europeen de Recherche et Formation Avancees en Calcul Scientifique
14	CSIRO-Mk3-6-0	Commonwealth Scientific and Industrial Research Organisation in collaboration with the Queensland Climate Change Centre of Excellence
15	FGOALS-g2	LASG, Institute of Atmospheric Physics, Chinese Academy of Sciences; and CESS, Tsinghua University
16	FGOALS-s2	LASG, Institute of Atmospheric Physics, Chinese Academy of Sciences; and CESS, Tsinghua University
17	FIO-ESM	The First Institute of Oceanography, SOA, China
18	GFDL-CM3	Geophysical Fluid Dynamics Laboratory
19	GFDL-ESM2M	Geophysical Fluid Dynamics Laboratory
20	GISS-E2-H	NASA Goddard Institute for Space Studies
21	GISS-E2-H-CC	NASA Goddard Institute for Space Studies
22	GISS-E2-R	NASA Goddard Institute for Space Studies
23	GISS-E2-R-CC	NASA Goddard Institute for Space Studies
24	HadGEM2-AO	Met Office Hadley Centre (additional HadGEM2-ES realizations contributed by Instituto Nacional de Pesquisas Espaciais)
25	HadGEM2-CC	Met Office Hadley Centre (additional HadGEM2-ES realizations contributed by Instituto Nacional de Pesquisas Espaciais)

26	INMCM4	Institute for Numerical Mathematics
27	IPSL-CM5A-LR	Institut Pierre-Simon Laplace
28	IPSL-CM5A-MR	Institut Pierre-Simon Laplace
29	MIROC5	Atmosphere and Ocean Research Institute (The University of Tokyo), National Institute for Environmental Studies, and Japan Agency for Marine-Earth Science and Technology
30	MIROC-ESM	Japan Agency for Marine-Earth Science and Technology, Atmosphere and Ocean Research Institute (The University of Tokyo), and National Institute for Environmental Studies
31	MIROC-ESM-CHEM	Japan Agency for Marine-Earth Science and Technology, Atmosphere and Ocean Research Institute (The University of Tokyo), and National Institute for Environmental Studies
32	MPI-ESM-LR	Max Planck Institute for Meteorology (MPI-M)
33	MPI-ESM-MR	Max Planck Institute for Meteorology (MPI-M)
34	MRI-CGCM3	Meteorological Research Institute
35	NorESM1-M	Norwegian Climate Centre

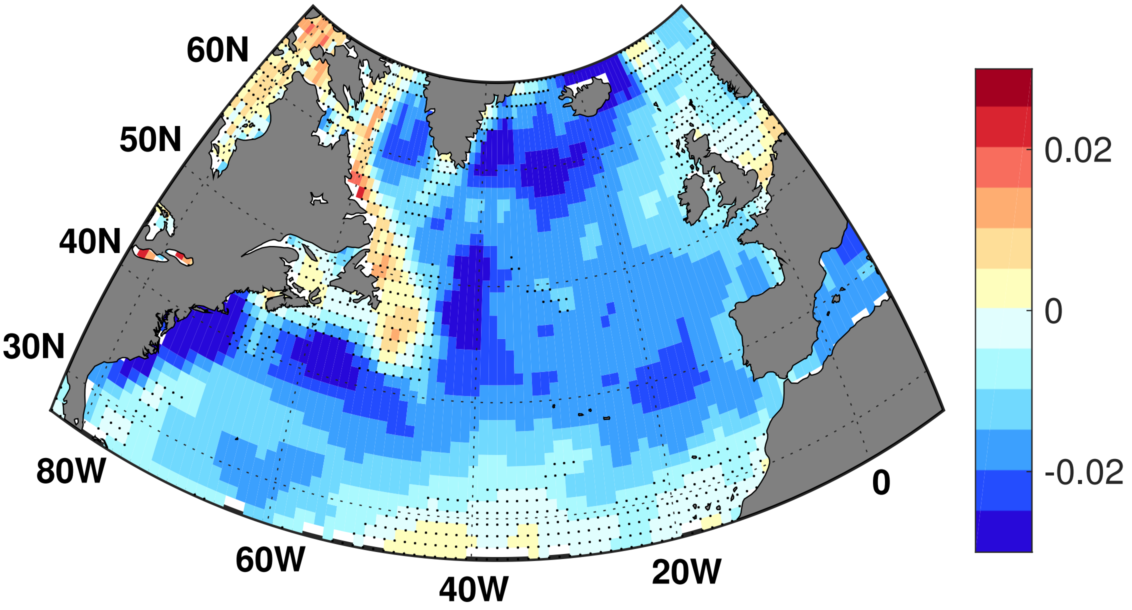

Supplementary Fig. 1. Annual mean sea surface temperature trends (Kyr^{-1}) from the satellite-based HadISST over the 36 years (1947-1982) prior to the analyzed period in the manuscript. The small black crosses show where the sea surface temperature trends are statistically insignificant based on a student t-test (p-value>0.05).

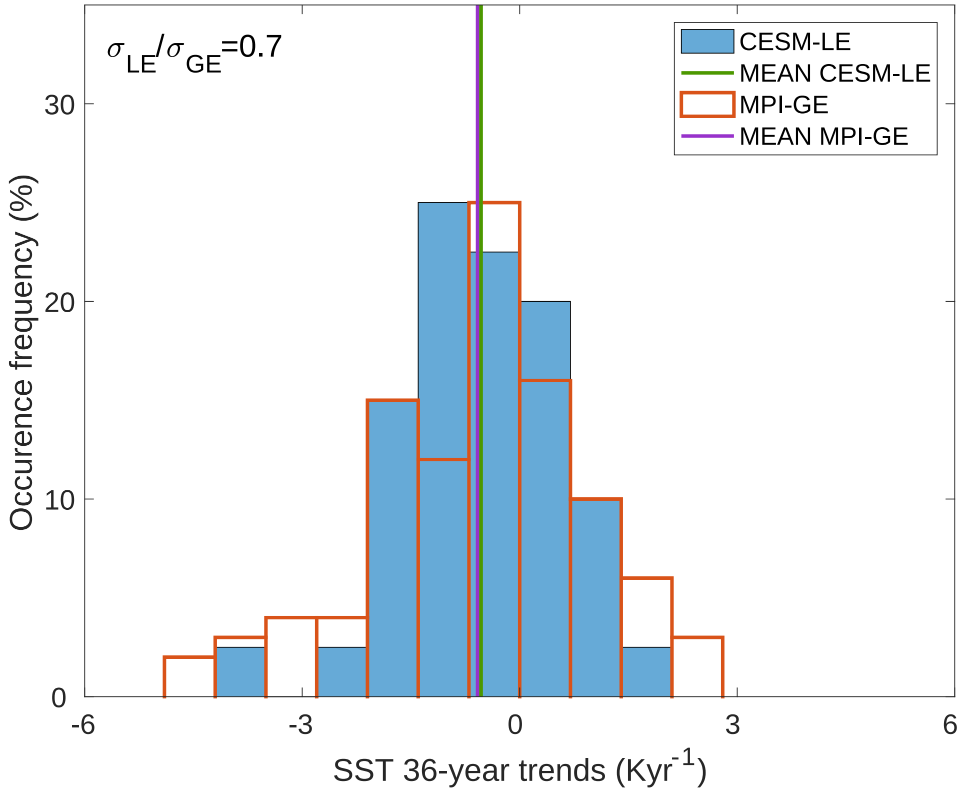

Supplementary Fig. 2. The occurrence frequency (in percentage) of 36-year (1982-2017) sea surface temperature trends (10^{-2}, Kyr^{-1}) over North Atlantic midlatitudes ($20°\mathrm{W} - 40°\mathrm{W}$ and $45°\mathrm{N} - 55°\mathrm{N}$) in the Community Earth System Model Large Ensemble (CESM-LE, blue) and Max Planck Institute for Meteorology Grand Ensemble (MPI-GE, red). The green and purple vertical lines show the mean of the CESM-LE and MPI-GE, respectively. The ratio between one standard deviation of the CESM-LE (σ_{LE}) and one standard deviation of MPI-GE (σ_{GE}) is shown in the top left corner: while CESM-LE may underestimate the variability of SST relative to MPI-GE, their forced response (vertical lines) is similar.

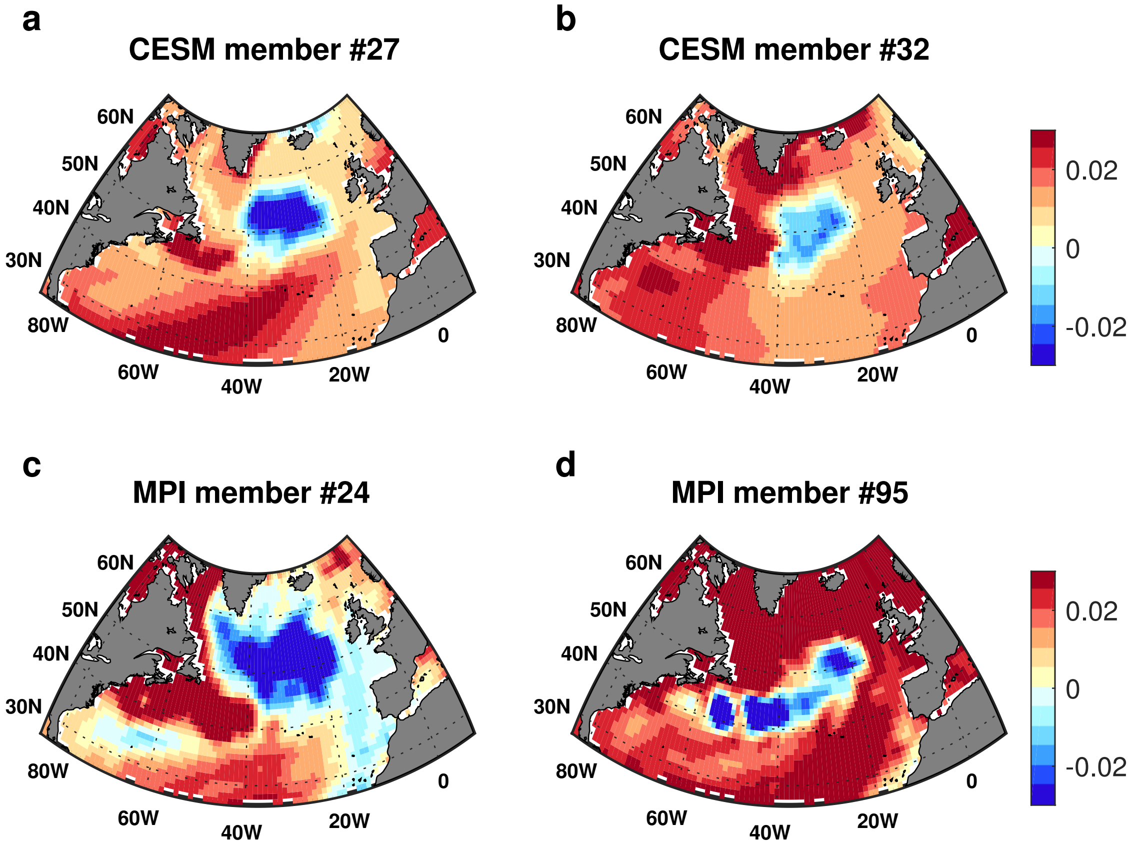

Supplementary Fig. 3. The 1982-2017 sea surface temperature trends (Kyr^{-1}) from a, member #27 in the Community Earth System Model Large Ensemble (CESM-LE), b, member #32 in CESM-LE, c, member #24 in the Max Planck Institute for Meteorology Grand Ensemble (MPI-GE), and d, member #95 in MPI-GE.

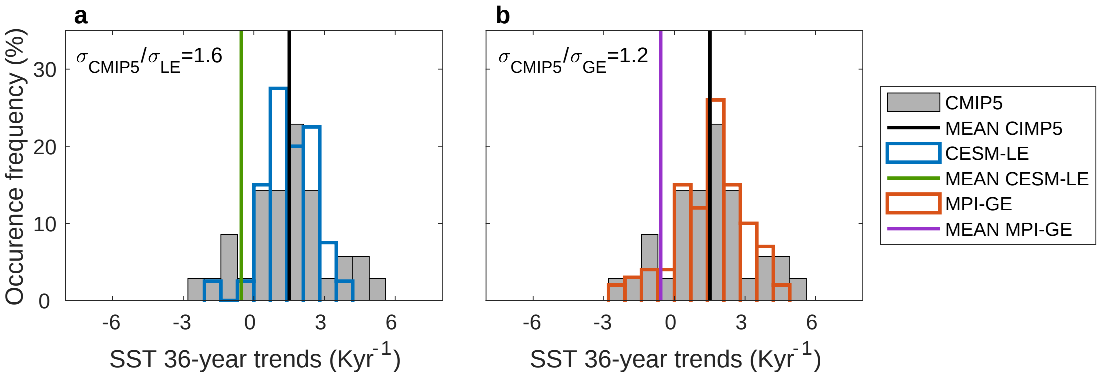

Supplementary Fig. 4. The occurrence frequency (in percentage) of 36-year (1982-2017) sea surface temperature trends (10^{-2}, Kyr^{-1}) over North Atlantic midlatitudes ($20°\mathrm{W} - 40°\mathrm{W}$ and $45°\mathrm{N} - 55°\mathrm{N}$) in the Coupled Model Intercomparison Project Phase 5 (CMIP5) models (gray) and (a) the Community Earth System Model Large Ensemble (CESM-LE) shifted around the mean of the CMIP5 (blue), and (b) Max Planck Institute for Meteorology Grand Ensemble (MPI-GE) shifted around the mean of the CMIP5 (red). The black, green and purple vertical lines show the mean of the CMIP5, CESM-LE and MPI-GE, respectively. The ratio between one standard deviation of the CMIP5 (σ_{CMIP5}) and one standard deviation of either the CESM-LE (σ_{LE}) or MPI-GE (σ_{GE}) are shown in each panel's top left corner. The different model formulations might account for $17\% - 38\%$ of the spread in North Atlantic midlatitudes sea surface temperature across the models.

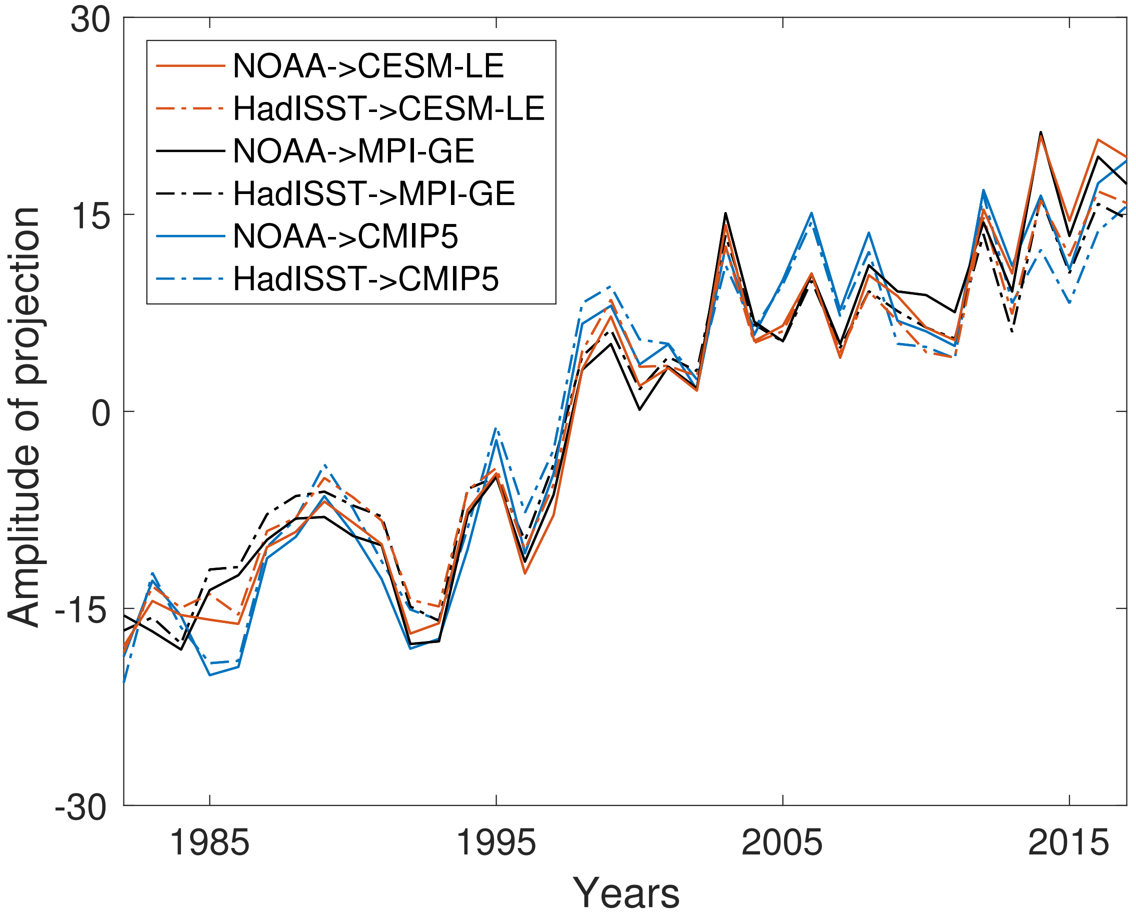

Supplementary Fig. 5. Projections of the North Atlantic sea surface temperature anomalies from satellite-based observations onto the simulated sea surface temperature fingerprints. Red, black and blue lines show the projections onto the leading modes from the mean of the Community Earth System Model Large Ensemble (CESM-LE), Max Planck Institute for Meteorology Grand Ensemble (MPI-GE) and Coupled Model Intercomparison Project Phase 5 (CMIP5), respectively. The solid and dashed lines show the projections using the NOAA and HadISST data sets, respectively.

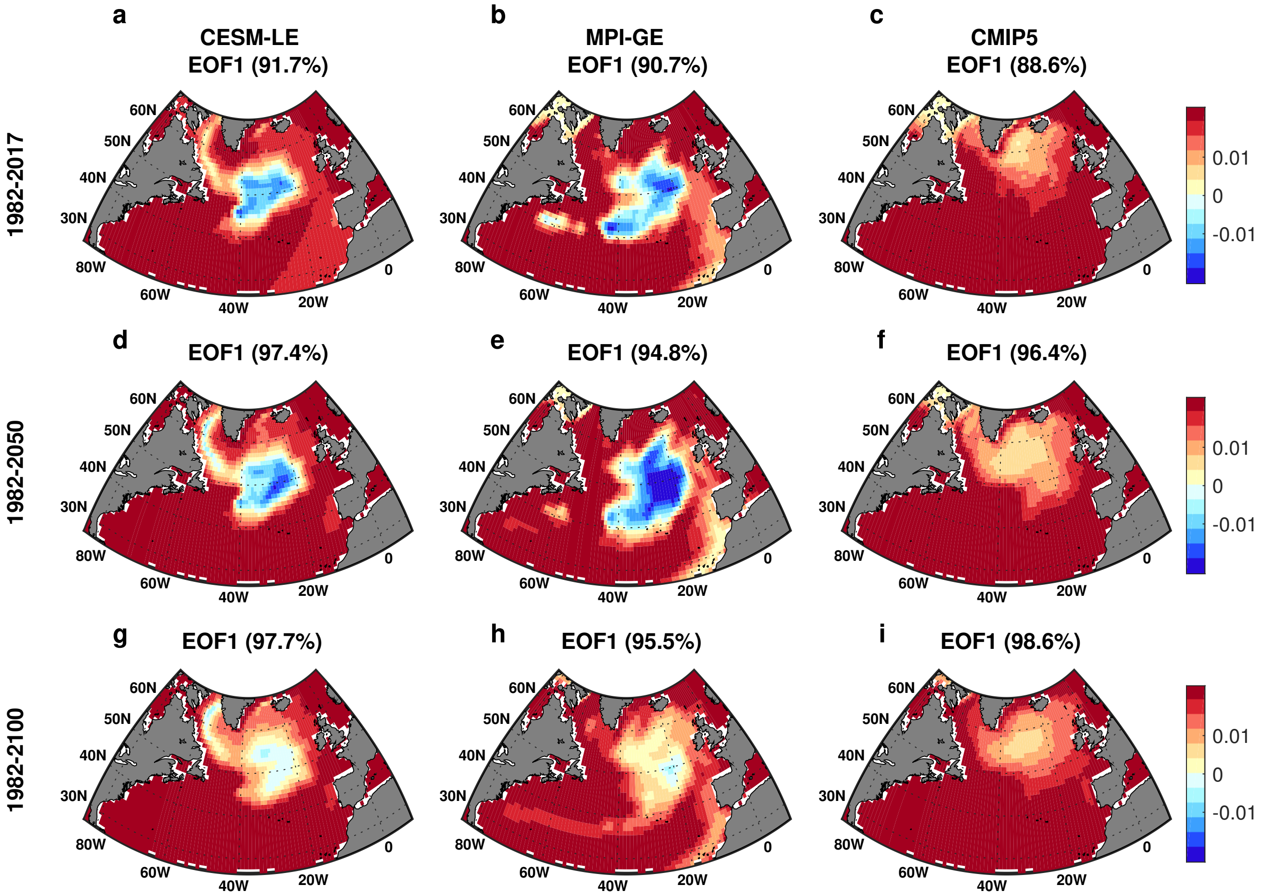

Supplementary Fig. 6. The leading mode (fingerprint) of the North Atlantic sea surface temperature over the (top row) 1982-2017 period, (middle row) 1982-2050 period, and (bottom row) 1982-2100 period. The modes are calculated using the mean of the (left column) Community Earth System Model Large Ensemble (CESM-LE), (middle column) Max Planck Institute for Meteorology Grand Ensemble (MPI-GE), and (right column) Coupled Model Intercomparison Project Phase 5 (CMIP5). The percentage in each panel represents the variability explained by each mode.
